# Geographic information system data from ambulances applied in the emergency department: effects on patient reception

**DOI:** 10.1186/s13049-016-0232-5

**Published:** 2016-03-31

**Authors:** Nikolaj Raaber, Iben Duvald, Ingunn Riddervold, Erika F. Christensen, Hans Kirkegaard

**Affiliations:** Research Department, Prehospital Emergency Medical Services, Central Denmark Region, Olof Palmes Alle 34, Aarhus N, DK-8200 Denmark; Research Center for Emergency Medicine, Aarhus University Hospital, Noerrebrogade 44, build. 30, Aarhus C, DK 8000 Denmark; Interdisciplinary Centre for Organizational Architecture (ICOA), Business and Social Sciences, Aarhus University, Bartholins Allé 12, Aarhus C, DK-8000 Denmark; Clinical Institute, Aalborg University, Sdr. Skovvej 15, Aalborg, DK-9000 Denmark; Emergency Department, Aarhus University Hospital, Norrebrogade 44, Aarhus C, DK-8000 Denmark; Department of Anesthesiology and intensive care medicine, Aalborg University Hospital, Hobrovej 18-20, Aalborg, DK-9000 Denmark

**Keywords:** GIS data, Trauma team activation, Medical emergency team activation, Emergency department organization, Emergency department nursing, Telemedicine

## Abstract

**Background:**

Emergency departments (ED) recognize crowding and handover from prehospital to in-hospital settings to be major challenges. Prehospital Geographical Information Systems (GIS) may be a promising tool to address such issues.

In this study, the use of prehospital GIS data was implemented in an ED in order to investigate its effect on 1) wait time and unprepared activations of Trauma Teams (TT) and Medical Emergency Teams (MET) and 2) nurses’ perceptions regarding patient reception, workflow and resource utilization.

**Methods:**

Intervention: From May 1st 2014 to October 31th 2014, GIS data was displayed in the ED. Data included real-time estimated time of arrival, distance to ED, dispatch criteria, patient data and ambulance contact information. Data was used by coordinating nurses for time activation of TT and MET involved in the initial treatment of severely-injured or critically-ill patients. In addition, it was used as a logistics tool for handling all other patients transported by ambulance to the ED.

Study design: The study followed a mixed-methods design, consisting of a quantitative study (before and after intervention) and a qualitative study (survey and interviews).

Participants: Participants included all patients received by TT or MET and coordinating nurses in the ED.

**Results:**

*1.) Quantitative*: 599 patients were included. The median wait time for TT and MET was 5 min both before and after the GIS intervention, showing no difference (*p* = 0.18). A significant reduction in the subgroup of waits >10 min was found (*p* < 0.05). No difference was found in unprepared TT and MET activations.

*2.) Qualitative*: Nurses perceived GIS data as a tool to optimize resource utilization and quality of all patients’ reception, critically or non-critically ill. No substantial disadvantages were reported.

**Discussion:**

The contradiction of measured median wait time and nurses perceived improved timing of team activation may result from having both RT- ETA and supplemental patient information not only for seriously-injured or critically-ill patients received by the TT and MET, but for all patients transported by ambulance. The reduction in waits > 10 minutes may have contributed to the overall perception of reduced wait time, as avoidance of long waits is clinically more important than reduction in the median wait time.

**Conclusion:**

A comparison of the use of prehospital GIS data in the ED with the control period showed no effect on median wait time for TT and MET, however, the number of waits of >10 min was reduced. On the other hand, nurses perceived implementation of GIS data as improving workflow, resource utilization and quality of all patients’ reception, critically as well as non-critically ill. There were no substantial disadvantages to the GIS application.

**Trial registration:**

ClinicalTrials.gov (NCT02188966).

**Electronic supplementary material:**

The online version of this article (doi:10.1186/s13049-016-0232-5) contains supplementary material, which is available to authorized users.

## Background

Emergency department (ED) crowding and handover from the prehospital to in-hospital settings are recognized challenges in EDs all over the world. Both crowding and patient handover are highly important issues to address in order to optimize quality and resource utilization in the ED [1–3].

Immediate and appropriate treatment is particularly important when dealing with seriously-injured or critically-ill patients. When expecting these patients in the ED, nurses activate ad-hoc Trauma Teams (TT) and Medical Emergency Teams (MET) to secure assessment and treatment immediately at the patient’s arrival. The timing of team activation is based on ambulance personnel’s subjective estimated time of arrival, as provided by phone to the ED. TT and MET are ad-hoc teams composed of select specialists from different departments, so time spent in the ED takes team members away from other in-hospital tasks. Therefore, optimal use of team resources may induce other in-hospital benefits.

ED personnel may also use estimated time of arrival (ETA) for non-critically ill patients. This patient group constitutes the majority of the population in the ED; in most cases, ambulance personnel do not announce when they are on their way to the ED with such patients. Nonetheless, valid information about these patients prior to arrival could optimize work in the ED. Prehospital geographical information system (GIS) data in the ED could provide a valid ETA for incoming patients, a tool for addressing the above-mentioned challenges. A search of the literature has revealed that no previous studies have systematically investigated the use of GIS data in the ED. Accordingly, this study had the following aims:

1. To examine the effect of GIS data information on wait time and unprepared activations of TT and MET (activation at or after the patient’s arrival) in the ED when receiving *seriously-injured* or *critically-ill patients.*
2. To explore the nursing staff’s subjective perception of GIS data implementation on a) wait time for TT and MET when handling *seriously-injured* or *critically-ill patients* and b) quality of patient reception, resource utilization, workflow, and work environment when handling *all patient categories* brought to the ED by ambulance.

## Methods

### Study design and setting

This study employed a mixed methods approach containing both quantitative and qualitative elements. The research followed the guidelines for reporting on a mixed methods study [4]. The quantitative study took highest priority of the two.

The study was conducted in the Horsens area of Central Denmark Region, Denmark. It involved regional ambulances and the ED at Regional Hospital Horsens (RHH). The size of Central Denmark Region is 13.142 km^2^ with a total population of 1.282.000, out of which the Horsens area constitutes approximately 208.000 people [5]. The demography of the area is mixed urban and rural with a population density between 55 and 159 per km^2^ [6].

In Central Denmark Region, the Emergency Medical Coordination Center (EMCC) prioritizes and coordinates all patient transport by ambulance. In case of accident or medical emergency, people call the emergency number 1-1-2. The call is forwarded to healthcare professionals (nurses, paramedics and physicians) at the EMCC who decide on an appropriate response according to the level of urgency.

RHH is one of five emergency hospitals in the region receiving trauma- and critically ill patients. The ED at RHH receives patients referred by general practitioners (GPs) and patients who have called 1-1-2. There is total number of 34.000 ED visits per year out of which 8000 patients are brought to the ED by ambulance, 4200 as GP referrals and 3800 after calling 1-1-2 (unpublished data 2014).

The TT or MET receive seriously-injured or critically-ill patients. The teams typically consist of eight to twelve health professionals (specialist physicians, radiologists, nurses and medical laboratory technicians), five to six of them coming from outside the department, all of whom stand by in the emergency room ready to attend to patients upon their arrival. In total, the TT and MET are activated approximately 650 times per year (unpublished data 2013). At RHH, a coordinating nurse makes the decision to activate the ED’s TT and MET following a trauma protocol and triage criteria based on information provided by prehospital ambulance personnel. The coordinating nurses are dedicated to the task of communicating with the ambulance personnel and other prehospital health professionals, such as GPs or physicians in prehospital mobile intensive care units. Teams are activated using a digital enhanced cordless telecommunications system (DECT). The notification was a text page including type of emergency call and where to go. The ETA was not included. According to protocol team members should come to bedside immediately.

### Control period

From May 1st 2013 to October 31th 2013, the ETA of incoming seriously-injured or critically-ill patients was provided by ambulance personnel or by physician-staffed mobile intensive care units. They provided the ETA by phone during patient transport to a coordinating nurse in the ED, and it was normally not updated en route. Generally, the call was made at the beginning of the transport, but was not specified by protocol. The timing of TT and MET activation was based on this ETA. Decision to activate and activation was preferably executed prior to patient arrival, but in some cases, the patient would arrive before team activation and activation would be done on arrival. If ETA was long nurses could delay team activation at their discretion. No ETA was provided for non-critically ill patients referred by a GP. For non-critically ill patients who had called 1-1-2, the ambulance personnel usually would call the coordinating nurse to provide information on the patient’s condition and ETA.

The control period was chosen to avoid seasonal differences. To our knowledge no changes in the prehospital or in-hospital organization was made in the time until the intervention period.

### Intervention period

From May 1st 2014 to October 31th 2014, prehospital GIS data was used to display real-time ETA (RT-ETA) of ambulances en route to RHH’s ED. Also displayed was supplemental patient information, including name, birth date and main dispatch criteria (e.g., unconsciousness, severe dyspnea, trauma). The phone number for the ambulance was also shown (Additional file 1). This information was available for all patients transported by ambulance to RHH’s ED, both non-critical and critically-ill patients. The GIS data was an add-on to the phone call from the ambulances and continued unaltered throughout the intervention period. The intervention period was preceded by a 1-month run-in period in which the ED staff was introduced to the GIS-data application and the aims of the study.

### Technical solutions

Real-time GIS data was delivered by a Simatech Enterprise System server collecting vehicle positions and transport status via a mobile network and the Terrestrial Trunked Radio system, TETRA (a digital radio network in Denmark called SINE). The data was extracted using a developed JAVA Enterprise Server application executed on a JBOSS application server platform. Data and GIS information was presented in a web application based on Google Maps and custom-developed real-time views. RT-ETA was based on distance in a beeline multiplied by a factor. Since the road network in Denmark is very fine-meshed, beeline approximations have empirically proved to be valid. The correction factor was determined by the urgency of the transport (lights and sirens) and adjusted to local conditions by analyzing transportations in the Horsens area on five different days of the week on different times of the day. For each vehicle, RT-ETA was updated every 10 s with adaptively increasing accuracy. The web application used to collect and display the GIS data in the ED is known as Dynamic Surveillance for Acute Medical Coordination. A standard DECT telephone provided by Ascom Denmark was used in the ED. GIS data was displayed using two standard desktop PCs and monitors in the ED’s receiving area. The hardware set-up was performed by an end-user. As the GIS application was web based, no additional software was required. The application was non-commercial and the EMCC Central Denmark Region provided access to the application.

### Quantitative study

#### Study design

Controlled before-after (CBA).

#### Participants

Participants included seriously-injured or critically-ill patients received by the TT or MET. Patients were identified by systematically searching all patient records at RHH in the study period for the specific activity codes relating to TT and MET activation. Activation criteria for TT and MET were the same for both periods.

#### Effect parameters

1) Wait time (Twait) was defined as the interval from TT or MET activation to patient arrival. Team activation automatically generated a time-log and registration for the type of team activated. Patients’ time of arrival was registered manually upon arrival at the ED. Time was registered similarly in the control and intervention period. We performed sub analysis of long waits, defined as waits > 10 min. 2) Unprepared TT and MET activation was identified as TT and MET activation after or at the patient’s time of arrival (Twait ≤ 0). The estimated time interval from team activation to arrival in the ED was 3 min. We therefore performed sensitivity analysis of team activations occurring within 3 min prior to the patient’s arrival at the emergency department or after patient arrival. Use of GIS-data was assessed irrelevant in activations occurring > 10 min after arrival, accordingly we performed analysis omitting these activations.

#### Distance and transportation time to the ED

Distance and transport time were determined using Google maps with the ambulance’s pick-up spot as the reference. To identify a patient’s pick-up spot, patient IDs obtained at the RHH’s ED were used to search the EMCC database in Aarhus. Thus, the pick-up spot was obtainable only if the patient ID had been registered by EMCC personnel at the time of ambulance dispatch.

### Qualitative study

An explorative qualitative approach was chosen to look at both critically and non-critically ill patients transported to the ED by ambulance. Participants included the coordinating nurses in the ED, and surveys and interviews were conducted in July/August and September 2014, respectively.

#### Survey

An anonymized web-based survey (SurveyMonkey®) was conducted, including nine questions concerning the use of GIS and its implications. A pilot survey had been tested among two ED nurses beforehand to assess the clarity and relevance of the questions. The modified survey was then distributed. Answers were rated using a five-point Likert scale. To increase the response rate, three reminders were sent out.

#### Interviews

Based on survey data, the most important topics were identified, and an interview guide was developed (Additional file 2). Four topics were identified: 1) perceptions of TT and MET timing, 2) perceptions of resource utilization and workflows, 3) perceptions of patient-reception quality, and 4) perceptions of work environment.

With the assistance of the head nurse, the coordinating nurses from RHH’s ED were invited to participate in the interviews. Participants were given written information about the study prior to the interview. A medical anthropologist with no prior association with the participants conducted semi-structured interviews using an interview guide. To generate more comprehensive data eliciting shared and/or dissimilar understandings and experiences with GIS data, the nurses were interviewed in pairs. The interviews lasted 35–55 min and were recorded and transcribed. The first and second author examined transcriptions independently. The literature was ordered according to the four topics and included only if there was agreement between the two examiners regarding their relevance and topic. Based on citations, the two examiners drew conclusions by consensus and reported on results.

### Statistics

For TT and MET wait time, a statistical power analysis for a two-sample means test was performed for sample size estimation. A 20 % reduction in wait time was estimated a clinically significant effect size, as a 20 % reduction would result in a 1 min decrease of wait time with a pre-intervention wait time of estimated 5 min. With alpha = 0.5 and power = 0.80, the maximum sample size needed was 64 in each group. Mann-Whitney U test was used for all non-parametric data. Chi 2 was used for binomial data. Two-sample t-test with equal variance was used on non-parametric data logarithmically transformed into normal distribution. A variance ratio test was used to test for equal variance. Data analysis was performed using STATA13. Data analysis of surveys was performed using Excel 2013. The level of significance was set at *p* ≤ 0.05.

### Ethics

The study was approved by the Regional Ethics Committee, Central Denmark Region. As a method study, it was exempted from the informed consent requirement (inquiry 180/2012). The Danish Data Protection Agency approved data handling (case no1-16-02-338-12). All data were anonymized.

## Results

### Quantitative study

#### Patients included

Three hundred six patients were registered as having been received by the TT or MET during the intervention period. In the control period, 293 patients were registered. 23 patients in 2014 and 13 in 2013 were excluded due to incorrect registration (no telephone log). In total, 563 patients were included.

#### Timing of TT & MET

**Fig. 1:**
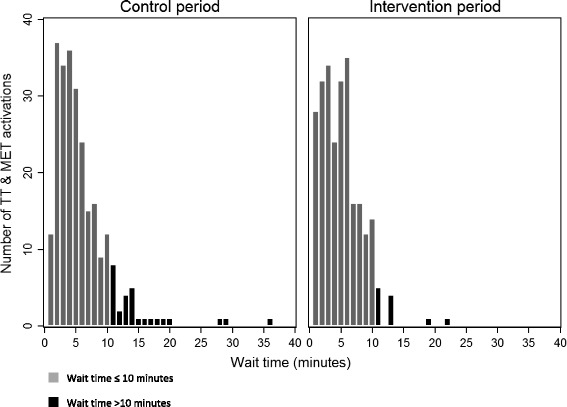
Distribution of wait time for Trauma Teams (TT) and Medical Emergency Team (MET) in the control period and after GIS data implementation in the intervention period

Table 1 summarizes the TT and MET wait time and unprepared activations of TT and MET divided in predefined time intervals. The median wait time for TT and MET before and after RT-GIS implementation was 5 min, showing no significant difference (*p* = 0.18). Table 2 depicts distance and time’s influence on wait time. For distance ≥ 20 km, a statistically significant reduction in wait time was found in the GIS group (*p* = 0.014). Since distance was not normally distributed logarithmic transformation and subsequent exponentiation was performed displaying a reduction of 1.4 min, 95 % CI [1.1; 1.7]. For all other strata, *p* ≥ 0.2. Figure 1 displays the wait time distribution with a tendency towards less variation in the intervention period. To quantify this, sub analysis of long waits was performed using a cut-off value of 10 min as the definition of a long wait. We found a significant reduction in the number of wait times of more than 10 min, from 11 % (*n* = 25) in the control period to 4 % (*n* = 11) in the intervention period, *p* < 0.05.

**Table 1:** TT and MET wait time and unprepared activations of TT and MET

	Control period	Intervention period	Total	*P*
	*N*	*N*	*N*	
TT & MET included	280	283	563	
Twait ≤0, (%)	26 (9.2)	29 (10.2)	53	0.70^a^
Twait ≤ 3, (%)	109 (38.9)	123 (43.5)	232	0.27^a^
Twait ≥ -10 ≤ 0, (%)	16 (5.9)	20 (7.3)	36	0.52^a^
Twait ≥ -10 ≤ 3, (%)	99 (36.7)	114 (41.6)	213	0.23^a^
Twait > 10, (%)	28 (11.0)	11 (4.0)	39	0.01^a^
	Median (iqr) [min]	Median (iqr) [min]		
Twait TT & MET	5 (3–8)	5 (3–7)		0.18^b^

*TT* trauma team, *MET* medical emergency team, *N* number of patients, iqr inter quartile range, *Twait* Wait time for TT & MET, *Unprepared activation* Time of team activation on or after patient arrival at the emergency department (Twait ≤ 0), *Twait ≤ 3* Time of team activation occurring within 3 min prior to the patient’s arrival at the emergency department or after patient arrival, *Twait ≥ -10* Sub analysis omitting team activations more than 10 min after arrival, *Twait > 10* Team wait time of more than 10 min

^a^Chi2

^b^Mann-Whitney

**Table 2:** Stratification of wait time (Twait) for TT and MET based on transportation time and distance from pick-up spot to ED

	Control period		Intervention period			
	*N*	*n* Missing (%)	*N*	*n* Missing (%)	*N* total	*P* ^a^
Patients included	157	123 (43 %)	230	53 (19 %)	387	
Distance, km [median (iqr)]		16 (3.8–25.7)		14.2 (4.1–24.9)		
Twait stratified by transportation time		Median (iqr) [min]		Median (iqr) [min]		
>0 < 5 min	45	5 (3–8)	66	4 (2–6)	111	0.33
≥5 < 20 min	49	4 (2–6)	95	5 (2–6)	144	0.57
≥20 min	88	5 (2.5–7)	113	4 (2–7)	201	0.14
Twait stratified by distance						
>0 < 5 km	45	4 (2–6)	66	4 (2–6)	111	0.90
≥5 < 19 km	47	5 (2–7)	80	4 (2–6.5)	127	0.88
≥20 km	66	5 (3–9)	85	4 (2–6)	151	0.03

*TT* trauma team, *MET* medical emergency team, *ED* emergency department, *N* number of patients, *iqr* inter quartile range; *n Missing* number of patients with missing data on transportation distance and transportation time, *Distance* distance from patient’s pick-up spot to ED

^a^Mann Whitney

A total of 29 (10 %) unprepared TT and MET were identified in 2014 and 26 (9 %) in 2013, showing no statistical significant difference. Moreover, we found no difference when looking at team activations executed within 3 min after patient arrival. Likewise, no difference was found when omitting team activations executed more than 10 min after patient arrival.

### Survey

#### Participants

Twenty-five coordinating nurses were invited to participate. Eleven (44 %) filled out the survey, and all (100 %) fully completed it.

#### Survey

**Fig. 2:**
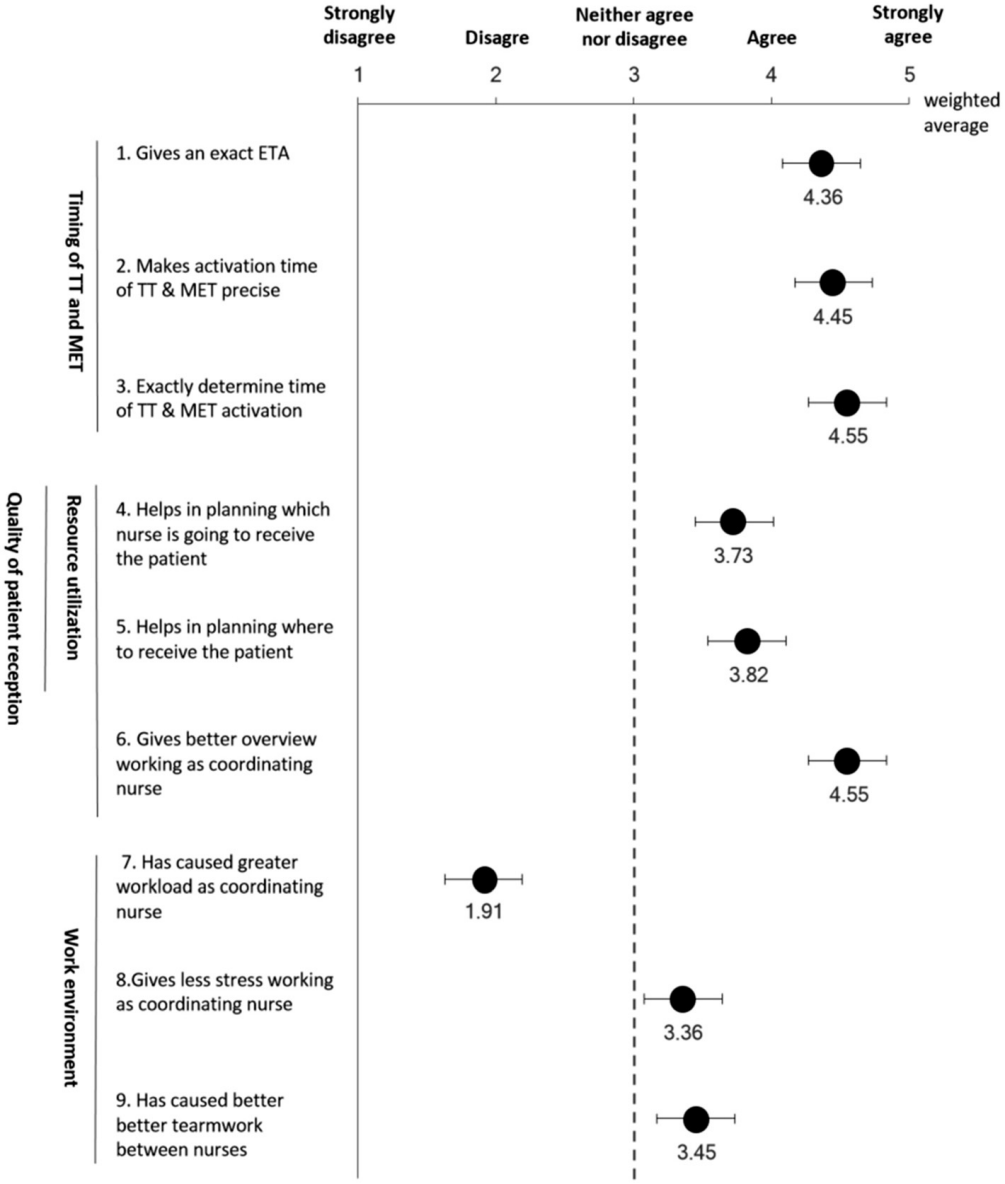
Survey among coordinating nurses; coordinating nurses were presented to the following statement: “How much do you agree or disagree with the following statements concerning the use of GIS data as a working tool? Error bars depict standard error

Figure 2 summarizes the results from the Survey among coordinating nurses related to predefined topics. With an average between four and five on the Likert-scale (agree to strongly agree), nurses perceived RT-ETA as precise, making activation of TT and MET more exact.

Furthermore, GIS data was considered helpful in planning which nurse would receive the patient and where, overall improving overview without regarding workload as having increased.

### Interviews

#### Participants

Four coordinating nurses (16 %) agreed to be interviewed [7]. Their years of experience as coordinating nurses ranged from under a year to more than 10 years.

##### Timing of TT and MET

TT and MET activation was perceived as more precise when using RT –ETA than when using ambulance personnel’s subjective ETA: “*…they [the ambulance personnel] can rarely say it [ETA] exactly, but here I can get it [ETA] rather exactly.*” Such perceptions reduced team members’ annoyance due to unnecessary wait times.

The RT-ETA provided for both critically and non-critically ill patients made it possible to predict patients’ ETA: “*…before we got this tool it was really hard to know when they [the patients] are actually coming.*”

##### Patient reception/handling

All together, GIS data implementation was regarded as improving patient treatment quality: “*…you can initiate a lot of things before the patient is actually at the hospital.*”

Participants regarded having the right ward and well-prepared staff as providing better patient reception. They believed that it was possible to avoid situations where non-critically ill patients were received by an unprepared staff.

##### Resource utilization and workflow

Availability of GIS data was perceived as having positive implications for coordinating patient reception and workflow. Having more information about patients on their way to the ED was perceived as a way to better organize and prepare. Meanwhile, participants noted that hasty patient transfers to other wards or departments were avoided, resulting in improved working relationships:

“*We get the right patient into the right ward to a greater extent…*”

“*…We can coordinate the use of wards and staff and competences that will be needed…*”

##### Work environment

Participants claimed that GIS data and supplemental patient information needed for planning work in the ED improved the work environment and decreased the nursing staff’s stress level: “*…it has influenced the level of stress (…) this [GIS] gives an overview which means that I feel more capable of handling the tasks…*”.

## Discussion

### Timing of TT and MET

No difference was found in the median wait time for TT and MET handling seriously-injured and critically-ill patients. The number of wait times of more than 10 min was significantly reduced in the intervention period. Also a minor reduction in mean wait time for transportation time >20 min was found. Nurses perceived the RT-ETA as being accurate, improving timing of team activation.

The contradiction of measured median wait time and perceived improved timing of team activation may result from having both RT- ETA and supplemental patient information not only for seriously-injured or critically-ill patients received by the TT and MET, but for all patients transported by ambulance, providing an overview and feeling of control. Interviews and surveys confirm this observation. Additionally, the reduction in long waits may have contributed to the overall perception of reduced wait time, as avoidance of long waits probably is clinically more important than reduction in the median wait time.

A US study demonstrated a 39 % underestimation of transport time provided by emergency medical technicians [8], and a Canadian study showed general underestimation of prehospital time, including transportation [9]. Findings that would result in longer wait time for teams therefore potentially enhance the effect of RT-ETA on TT and MET wait time in the ED. To our knowledge, this is the first study evaluating the effect of GIS data implementation on patient handling in the ED. Researchers commonly assume that having a reliable ETA for incoming ambulances diminishes unnecessary wait times and avoid unpreparedness in the ED [10]. Our study confirms this assumption when looking at number of avoided long waits, but not with regard to median wait time. At the same time, no substantial disadvantages for GIS data implementation in the ED were found.

### Patient reception/handling quality

The quantitative effect parameter was the measured rate of unprepared TT and MET activations, which was not reduced by RT-ETA availability. In contrast, the nursing staff reported improved patient reception. The possible reasons for this discrepancy mirror those described for the timing of TT and MET. Earlier studies have demonstrated a tendency for ambulance personnel to underestimate ETA [8] at least on short runs [11]. An underestimation results in earlier TT and MET activation. Underestimation of ETA will make unprepared team activations more unlikely, making a further reduction in unprepared activations difficult to achieve.

### Strengths and limitations

The quantitative evaluation of wait time was conducted as a before and after study. Our measure of team members wait time (Twait) was a surrogate, since the team’s actual arrival time in the ED was not registered. Our measure was based on the time difference between team activation and the patient’s arrival time. We find this wait time an adequate surrogate since protocol prescribed team members to come to bedside immediately after notification minimizing team members’ delay. Thus, the adequate wait time measurement and inclusion of the required number of participants gave the results both internal validity and generalizability.

RHH’s geographical and demographical setting is typical for medium-sized hospitals in Denmark, but the effect of GIS data implementation in an ED may differ in another setting, whether more urban, rural or differently-sized. The RT-ETA in our study was adjusted to the local geographical setting but not systematically validated. Nevertheless, as the RT-ETA was continuously updated with increasing accuracy and team-activation would occur when distance to the ED was short, these inaccuracies would gradually be compensated for. Consequently, the potential inaccuracies of the RT-ETA would probably not affect timing of team activation. Timing of team activation was decided by the coordinating nurses at their discretion, which could have resulted in both longer and shorter wait time depending on the individual nurse. The reliability of distance and transportation time’s influence on wait time was negatively affected by the high rate of missing data in 2013 (Table 2).

The qualitative approach provided analytical insight into implications for the nursing staff, which would not have been possible following a quantitative approach alone.

The small number of nurses interviewed limits the conclusions to be drawn from this portion to being theme generating rather than definitive. The survey response rate of 44 % may have resulted in nonresponse bias. We have no data describing nonrespondents in order to account for any differences compared to the respondents.

The study design did not include sham GIS data and having just any random ETA in contrast to nothing might have resulted in the nurses’ positive attitude towards the GIS data implementation. However, 6 months with inaccurate ETA would probably had given rise to obvious problems when planning patients’ reception and not resulted in the positive perception displayed in both survey and interviews.

The staff’s knowledge of the project’s objectives may have improved their performance (Hawthorne effect) contributing to an overestimation of the GIS data application’s benefits.

## Conclusions

GIS data implementation in the ED did not reduce median wait time for the TT and MET however, the number of waits of more than 10 min was reduced. There was no observed reduction in unprepared TT and MET activations. At the same time, during the reception of both critically and non-critically patients, ED nursing staff perceived GIS data as a tool for optimizing staff and ward utilization in the ED, improving workflow, augmenting the quality of patient treatment, and ameliorating the work environment. No substantial disadvantages to GIS data implementation were found.

## Additional files

Additional file 1: Screen shot of GIS application used in Regional Hospital Horsens. (PDF 396 kb) Additional file 2: Interview guide used for semi-structured interviews with coordinating nurses at RHH’s ED. (PDF 14 kb)
