# Supplementary material for: Geographic information system data from ambulances applied in the emergency department: effects on patient reception
Source: Scand J Trauma Resusc Emerg Med. 2016 Mar 31;24:39. doi: 10.1186/s13049-016-0232-5 (PMC4815218; doi:10.1186/s13049-016-0232-5)
Supplement: Additional file 1: — Screen shot of GIS application used in Regional Hospital Horsens. (PDF 396 kb) [file 13049_2016_232_MOESM1_ESM.pdf]

**Appendix 1.** GIS data web application, screenshot (English simulation)

**A**

| Type | Amb | Mobile / TETRA | Level | ETA | Dispatch code | Description | From/To address | Primary notification | Name | Surname | PI number |
|---|---|---|---|---|---|---|---|---|---|---|---|
| | 3456 | M:51563518<br>T:5442090 | A | Hand over OK | Traffic accident _possible high energy trauma | | F:MTV S E45 M60 133<br>T: MHOR ED, entrance F (Closed) | Received from 1-1-2 14:06 Text: Site of damage: Two others with head pain. Everybody wearing safety belt. One car crashed into another | [illegible] | [illegible] | [illegible] |
| | 3451 | M:51563514<br>T:5442070 | A | Hand over OK | – | Is in outpatient clinic no. 3 | F:MTV S E45 M60 133<br>T: MHOR ED, entrance F (Closed) | Received from 1-1-2 14:06 Text: Site of damage: Two other with head pain. Everybody wearing safefy belt. One car crashed into another | | | |
| | 3448 | M:5442060<br>5442061 | A | Hand over OK | Planned transport _ Transport directly to hospital. Unstable vital signs | Possible MI. Previous heart disease | F:KIRKEGADE 18,ULDUM<br>T:MHOR M2 | EMCC: 385 DISP: RM1047 Comm: Possible MI, previous heart disease no further info. Prehospital physician on site | [illegible] | [illegible] | [illegible] |

**D**

| Type | Amb | Mobile / TETRA | Level | ETA | Dispatch code | Description | From/To address | Primary notification | Name | Surname | PI number |
|---|---|---|---|---|---|---|---|---|---|---|---|
| | 4166 | M:5446090<br>5446091 | D | 132.4 min.<br>110.3 km | – | Phone ETA to Frederikshavn - 97641993 | F:FREDERIKSHAVN Hospital<br>T: MHOR ED | From dep. M2 4th floor. After admission in Frederikshavn, now further treatment on home hospital. Treated for Erysipelas and DVT. | [illegible] | [illegible] | [illegible] |
| | 4159 | M:5446030<br>5446031 | D | 18.2 min.<br>15.2 km | – | Pneumonia | F:SPIREAVEJ 12,GALTEN<br>T: MHOR ED, entrance F (Closed) | EMCC: 385 DISP: RM5271 Comm: Pneumonia. D# | [illegible] | [illegible] | [illegible] |
| | 3443 | M:51563504<br>T:5442040 | D | Pt. contact | – | Community nurse 22103686 Possibly bleeding ulcer | F:GAMMEL JERNBANEGADE 6,HORSENS<br>T:MHOR ED | EMCC: 385 DISP: RM1015 C omm: Community nurse 22103686, possibly bleeding ulcer | [illegible] | [illegible] | [illegible] |

Type: Ambulance type; AMB: Ambulance number; Mobile/TETRA: Phone/radio number; Level: Level of urgency; ETA: Estimated Time of Arrival
