# Supplementary material for: Geographic information system data from ambulances applied in the emergency department: effects on patient reception
Source: Scand J Trauma Resusc Emerg Med. 2016 Mar 31;24:39. doi: 10.1186/s13049-016-0232-5 (PMC4815218; doi:10.1186/s13049-016-0232-5)
Supplement: Additional file 2: — Interview guide used for semi-structured interviews with coordinating nurses at RHH’s ED. (PDF 14 kb) [file 13049_2016_232_MOESM2_ESM.pdf]

Appendix 2 - Interview guide used for semi structured interviews with coordinating nurses (English translation)

Research question	Interview questions	Elaborating questions	
How do coordinating nurses assess GIS data as a working tool?	How is GIS data used in the ED? Please give specific examples.<br><br>Combined with an exercise with picture of screenshot depicting the GIS-application.<br><br>What do you see from this picture and how do you use the different information?	a) What is the influence of GIS data on planning which nurse should receive the patient?<br>b) What is the influence of GIS data on planning where to receive the patient?	**Resource utilization**
		c) Is GIS data useable in determining when MET or TT should be activated?	**Timing of TT &MET**
	Are there any advantages/disadvantages by using GIS data? Keep discussing till it is clear where you agree or disagree.	a) Does TT or MET show up later in the ED?	
		b) Is reception of the patient improved? Does the patient meet the right competence faster?	**Quality of patient reception/ handling**
	Has implementation of GIS data changed working routines? What did you do before? Please give examples.		
	What is the most and the least useful about GIS data?		
	You have told how you use GIS data, but could it be utilized in other ways?		
How does the use of GIS data influence work environment, according to the nurses?	Has GIS data influenced you work environment? Keep discussing till it is clear where you agree or disagree.	a) When working as coordination nurse, is the level of stress influenced? If yes, how?<br>b) Is the teamwork in the department?	**Work environment**
How is the design of the GIS-application evaluated by the nurses?	Have you experienced inaccurate or wrong information? Please give examples.	a) What implications could it have?	
	Are there any information you are missing or ways the application could be improved?		
